# Supplementary material for: Systematic Evaluation of Parameters Important for Production of Native Toxin A and Toxin B from Clostridioides difficile
Source: Toxins (Basel). 2021 Mar 27;13(4):240. doi: 10.3390/toxins13040240 (PMC8066640; doi:10.3390/toxins13040240)
Supplement: Supplementary file 1 [file toxins-13-00240-s001.pdf]

Supplementary Materials: Systematic Evaluation of Parameters Important for Production of Native Toxin A and Toxin B from *Clostridioides difficile*

Aria Aminzadeh and René Jørgensen

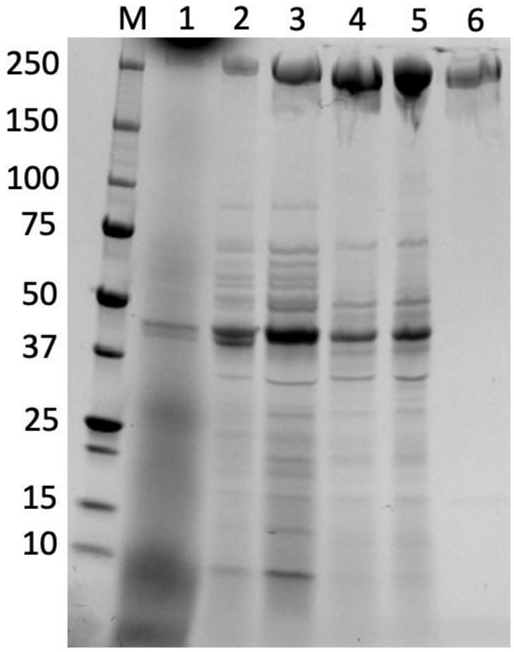

Figure S1. SDS-PAGE showing purity of TcdA after each purification step. lane M: molecular weight markers (kDa), lane 1: filtered culture supernatant (stage 1), lane 2: diafiltrated culture supernatant containing TcdA and TcdB (stage 2), lane 3: TcdA after Q Sepharose (stage 3), lane 4: TcdA after MonoQ (stage 4), lane 5: TcdA after ultrafiltration/concentration (stage 5), lane 6: TcdA after Superdex 200 (stage 6). Purity of TcdA was estimated from the SDS-PAGE using Image Lab 6.1 software (Bio-Rad, Hercules, CA, USA, 2019). after each purification step and is as follows; stage 2: 7.9%, stage 3: 34%, stage 4: 72.5%, stage 5: 72.5%, stage 6: >99%.

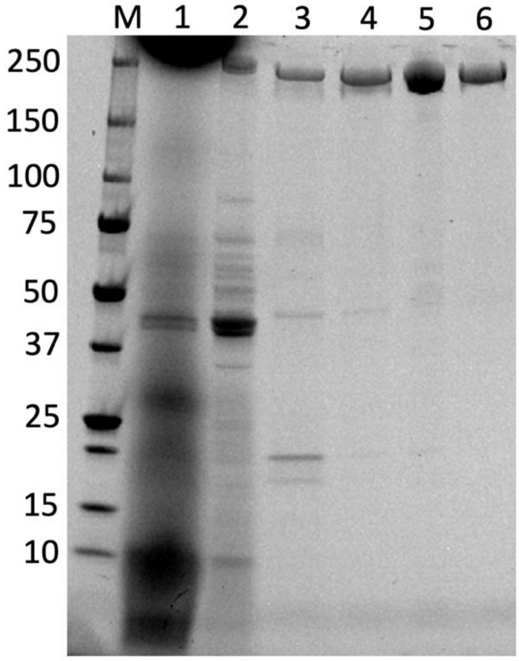

Figure S2. SDS-PAGE showing purity of TcdB after each purification step. lane M: molecular weight markers (kDa), lane 1: filtered culture supernatant (stage 1), lane 2: diafiltrated culture supernatant containing TcdA and TcdB (stage 2), lane 3: TcdB after Q Sepharose (stage 3), lane 4: TcdB after MonoQ (stage 4), lane 5: TcdB after ultrafiltration/concentration (stage 5), lane 6: TcdB after Superdex 200 (stage 6). Purity of TcdB was estimated from the SDS-PAGE using Image Lab 6.1 software after each purification step and is as follows; stage 2: 3.9%, stage 3: 65.6%, stage 4: 95.2%, stage 5: 98%, stage 6: >99%.

Table S1. TY^{opt} supports increased toxin production in *C. difficile* strain VPI 10463.

Culture Medium	*C. difficile* Strain	pH	OD_{600}	TcdB (ng/mL)
TY^{opt}	R20291	7.5	0.98	3980 (3660–4851) (n = 6)
TY^{opt}	VPI 10463	7.5	1.83	3757 (3557–4434) (n = 3)

All cultures were grown in 50 mL TY^{opt} media and incubated unagitated for 3 days at 37 °C with OD_{600} measured on day 3.

Table S2. The effect of various HEPES and NaCl concentrations on the thermal stability of TcdA.

HEPES (mM)	NaCl (mM)	T_m (°C)
50	0	48.2
50	125	51.4
50	250	52.8
50	500	53.7
50	750	53.7
50	1000	54.0
100	0	48.9
100	125	51.3
100	250	52.5
100	500	53.5
100	750	53.6
100	1000	53.4
200	0	49.6
200	125	51.2
200	250	52.2
200	500	53.8
200	750	54.5
200	1000	54.0
300	0	50.0
300	125	51.6
300	250	52.6
300	500	53.9
300	750	54.4
300	1000	54.9

Thermal stability was measured by DSF using real-time PCR. HEPES was adjusted to pH 7.5 for all conditions.

Table S3. The effect of various salts added to 100 mM HEPES on the thermal stability of TcdA.

Salt	Concentration (mM)	T_m (°C)
NaCl	100	51.1
NaCl	500	52.8
NH_4Cl	100	51.7
NH_4Cl	500	52.5
$MgSO_4$	100	53.7
$MgSO_4$	500	54.6

Thermal stability was measured by DSF using real-time PCR. TcdA was buffered in 100 mM HEPES (pH 7.5) for all conditions.

Table S4. The effect of $MgSO_4$ and glutamic acid on the thermal stability of TcdA.

$MgSO_4$ (mM)	Glutamic Acid (mM)	T_m (°C)
0	0	49.5
250	0	54.0
500	0	54.1
0	250	52.8
0	500	54.6
250	250	55.4
250	500	56.5
500	250	53.9
500	500	56.7

Thermal stability was measured by DSF using real-time PCR. TcdA was buffered in 100 mM HEPES (pH 7.5) for all conditions.

Table S5. The effect of various HEPES and NaCl concentrations on the thermal stability of TcdB.

HEPES (mM)	NaCl (mM)	T_m (°C)
50	0	46.7
50	125	51.5
50	250	51.6
50	500	51.0
50	750	50.2
50	1000	49.7
100	0	49.0
100	125	51.4
100	250	51.4
100	500	51.2
100	750	51.2
100	1000	49.7
200	0	50.2
200	125	51.2
200	250	51.1
200	500	51.2
200	750	50.3
200	1000	49.6
300	0	50.7
300	125	51.1
300	250	50.3
300	500	49.7
300	750	48.8
300	1000	48.9

Thermal stability was measured by DSF using real-time PCR. HEPES was adjusted to pH 7.0 for all conditions.

Table S6. The effect of various salts added to 100 mM HEPES on the thermal stability of TcdB.

Salt	Salt Conc (mM)	T_m (°C)
NaCl	250	51.9
NaCl	500	51.5
NH_4Cl	250	50.8
NH_4Cl	500	49.4
$MgSO_4$	250	49.7
$MgSO_4$	500	49.5
$NaSO_4$	100	52.5

Thermal stability was measured by DSF using real-time PCR. TcdB was buffered in 100 mM HEPES (pH 7.0) for all conditions.

Table S7. The effect of $NaSO_4$ and glutamic acid on the thermal stability of TcdB.

$NaSO_4$ (mM)	Glutamic Acid (mM)	T_m (°C)
0	0	45.2
250	0	52.1
0	250	52.1
0	500	52.9
250	250	53.1
250	500	52.9

Thermal stability was measured by DSF using real-time PCR. TcdB was buffered in 100 mM HEPES (pH 7.0) for all conditions. .
